# Abiraterone acetate and docetaxel with androgen deprivation therapy in high-volume metastatic hormone-sensitive prostate cancer in China: an indirect treatment comparison and cost analysis

**DOI:** 10.1186/s12962-019-0193-4

**Published:** 2019-12-13

**Authors:** Xin Hu, Shuli Qu, Xingxing Yao, Chaoyun Li, Yanjun Liu, Jianye Wang

**Affiliations:** 1 Department of Pharmacy, Beijing Hospital, Beijing, China; 2 Real World Solutions, IQVIA, Shanghai, China; 3 Health Economics & Outcome Research, Sanofi, Shanghai, China; 4 Department of Urology, Beijing Hospital, No. 1 Dongdan Dahua Road, Dongcheng District, Beijing, 100730 China

**Keywords:** Prostate cancer, Docetaxel, Abiraterone, Cost analysis

## Abstract

**Background:**

To conduct an indirect treatment comparison of patients with high-volume mHSPC and a cost analysis between Abi-ADT and Doc-ADT therapies in China.

**Methods:**

The Bucher technique for indirect treatment comparison was used. A cost analysis was conducted from both healthcare and patient perspectives.

**Results:**

The indirect treatment comparison demonstrated no significant difference in PFS for Abi-ADT versus Doc-ADT (HR: 0.84, 95% CI 0.66–1.07). Doc-ADT therapy costs less than Abi-ADT, with potential savings of up to RMB 887,057 per patient from the healthcare perspective and RMB 226,210 per patient from the patient perspective.

**Conclusions:**

No significant differences in PFS between Doc-ADT and Abi-ADT therapy for patients with high-volume mHSPC. Doc-ADT therapy is a cost-saving alternative to Abi-ADT in China.

## Background

Prostate cancer is the most common cancer for men worldwide [1]. It also ranks fifth and tenth in estimated deaths worldwide and in China, respectively [2]. The incidence rates of prostate cancer within the Asian population have historically been lower than those in the Western population; however, in recent years, the incidence and mortality rate in China have grown rapidly, with a three-fold increase in the last decade. This dramatic rise in incidence has led to 25,000 deaths annually with an estimated 5-year survival rate of 54% [3, 4].

In China, due to the low rate of prostate-specific antigen screening to detect cancer at an early stage, a panel of local clinical experts reported that most Chinese patients at the time of diagnosis had high-volume metastatic hormone-sensitive prostate cancer (mHSPC), which included the presence of visceral metastases, a bone-metastasis burden categorised by site (beyond the axial skeleton) or by a high number of lesions, or a combination of these [5].

The treatment for men with mHSPC has historically included androgen deprivation treatment (ADT), which can be accomplished by surgical castration or medical suppression of testicular function with synthetic analogues of gonadotropin-releasing hormone [6]. The addition of docetaxel or abiraterone acetate plus prednisone to ADT has been recommended for patients with newly diagnosed mHSPC [7]. The CHAARTED [5], LATITUDE [8] and GETUG-AFU-15 [8, 9] trials demonstrated that the addition of docetaxel and abiraterone acetate to ADT improved overall survival (OS) and progression-free survival (PFS) among men with mHSPC compared with ADT alone. However, no head-to-head clinical trials have compared abiraterone acetate plus prednisone/prednisolone with ADT (Abi-ADT) to docetaxel with ADT (Doc-ADT). A recently published network meta-analysis (NMA) conducted by Wallis in 2017 revealed no significant difference in OS for men treated with Abi-ADT versus Doc-ADT. However, the pooled result of PFS was not reported. A growing belief among the oncology community is that delaying the progression of metastatic disease is a worthwhile goal, even without improvement in OS [10]. Based on the above rationale, the Wallis study must be updated with the PFS outcomes associated with Doc-ADT and Abi-ADT for patients with high-volume mHSPC. With rapidly increasing healthcare demands and limited medical resources in China, medical institutions are confronted with the urgent task of controlling medical expenses. Chinese national level medical spending exceeded significantly that of all G7 members except the US in terms of current purchase power parity [11]. In addition, there is an ongoing public debate about the effective introduction and spreading of value based medicine concepts, and introduction of cost-effectiveness criteria into official policy making in most world regions [12]. Therefore, the application of Doc-ADT and Abi-ADT therapies should be carefully considered by balancing clinical outcomes and related costs. No economic data comparing these two therapies in China are available.

In summary, the main objective of our study is to determine the comparative efficacy and costs of Doc-ADT and Abi-ADT therapies in the treatment of patients with high-volume mHSPC. To achieve this goal, we first updated the NMA outcomes of the two treatments. We then performed an economic evaluation comparing Doc-ADT and Abi-ADT therapies in China through a cost analysis from both healthcare and patient perspectives.

## Indirect treatment comparison

### Identification of eligible trials and data extraction

Studies of adults with high-volume mHSPC comparing the efficacy of Doc-ADT or Abi-ADT to ADT alone were identified. Only randomised controlled trials (RCTs) were included. PubMed and Cochrane Library were searched for trials published in English, and the Chinese databases CNKI and WanFang were searched for studies published in Chinese. Search terms were derived from the Wallis study and updated from August to November 2017. Table 1 summarises the PICOS (population, intervention, controls, outcomes, and setting) elements corresponding to the research questions. The primary endpoints were OS and PFS.

**Table 1:** PICOS statement for the inclusion and exclusion criteria

	Inclusion	Exclusion
Study population	Patients with high-volume metastatic hormone-sensitive prostate cancer, regardless of age, sex, ethnic group or disease status	Any not listed in the inclusion criteria
Intervention	Docetaxel + ADT	Any not listed in the inclusion criteria
Comparator	Abiraterone + prednisone + ADT ADT alone	Any not listed in the inclusion criteria
Outcome measures	Clinical efficacy outcomes Safety outcomes	Any not listed in the inclusion criteria
Study design	Randomised clinical trials (RCTs)	Editorials OR Notes OR Comments OR Letters OR Case reports OR Pharmacokinetic studies OR Epidemiology studies
Restrictions	Full-text published manuscripts in English or Chinese Year limitation: up to Nov 2017	Duplicates Not full-text published manuscripts Non-English or non-Chinese studies

Two reviewers (SQ and YL) independently extracted all data from the eligible trials. For each included trial, we extracted the characteristics of the participants and the interventions, efficacy and safety outcomes, the sample size (randomised and analysed) in each arm, numerical results, and loss to follow-up data.

We assessed the risk of bias in each included trial using the Cochrane Collaboration’s Risk of Bias tool [13].

### Statistical methods

The Kaplan–Meier (KM) survival data of Doc-ADT and Abi-ADT were reconstructed by applying Guyot’s method [14], which uses digital software to read in the coordinates of the KM curves from the published graph and uses the information on numbers at risks, often published at four or five time points under the x-axis of the KM graph, and total number of events, to reconstruct the Kaplan–Meier data. We used this method to reconstruct the KM survival data for Doc-ADT and Abi-ADT arms from the corresponding KM survival curves in CHAARTED [15] and Fizazi’s [8] trials, respectively.

The hazard ratio (HR) of response to Doc-ADT and Abi-ADT versus ADT alone was calculated independently using Review Manager 5.3 (The Nordic Cochrane Centre, The Cochrane Collaboration, Copenhagen, Denmark) software. A random effect model was used due to the clinical heterogeneity inherent in the data. Heterogeneity among the studies was assessed using Chi square and *I*^*2*^ statistics. The pooled results are presented in the form of forest plots.

An indirect treatment comparison was performed to assess the clinical effects of Doc-ADT and Abi-ADT using ADT as the common comparator arm. We used Bucher’s successive pairwise approach [16], which has been identified as an appropriate method for performing indirect treatment comparisons of HRs [17]. The indirect treatment comparison was conducted using Excel 2013 (Microsoft Inc, Richmond, WA, USA).

### Indirect treatment comparison results

#### Evidence synthesis

Among 235 identified studies, 4 papers from 3 clinical trials were eligible for inclusion: the CHAARTED [5, 15], LATITUDE [8], and GETUG-AFU15 trials [9]. Patients in the LATITUDE trial generally had high-volume mHSPC as 96% of the patients had bone lesions with Gleason scores ≥ 8 and ≥ 3. Compared with the Wallis study, we also extracted the PFS data of patients with high-volume mHSPC from these 4 papers.

Overall, 1895 patients with high-volume mHSPC were included in the analysis; 354 (18.7%) patients received Doc-ADT, 597 (31.5%) received Abi-ADT, and 944 (49.8%) received ADT alone.

#### Characteristics and quality of the included studies

**Fig. 1:**
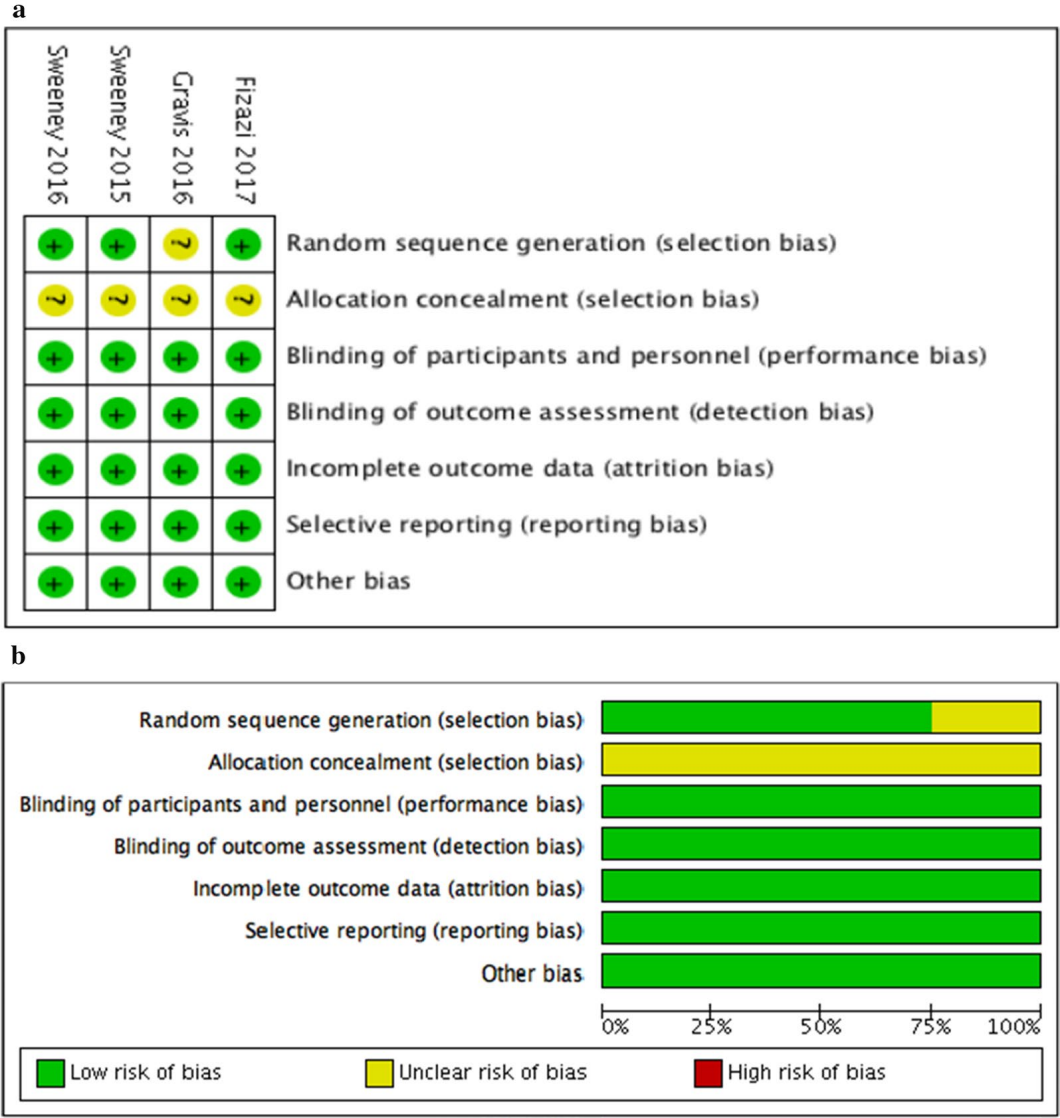
Risk of bias assessment of the included studies: **a** risk of bias graph and **b** risk of bias summary

In the Doc-ADT trials (CHAARTED and GETUG-AFU 15), the patients in the experimental arm received docetaxel 75 mg/m^2^ for a maximum of six [15] or nine [9] cycles. In the Abi-ADT trial (LATITUDE), the patients in the experimental arm received abiraterone acetate 1000 mg/d with prednisone 5 mg/d [8]. The quality of all included studies was assessed, and a low risk of bias with adequate randomisation was indicated (Fig. 1).

#### OS

**Fig. 2:**
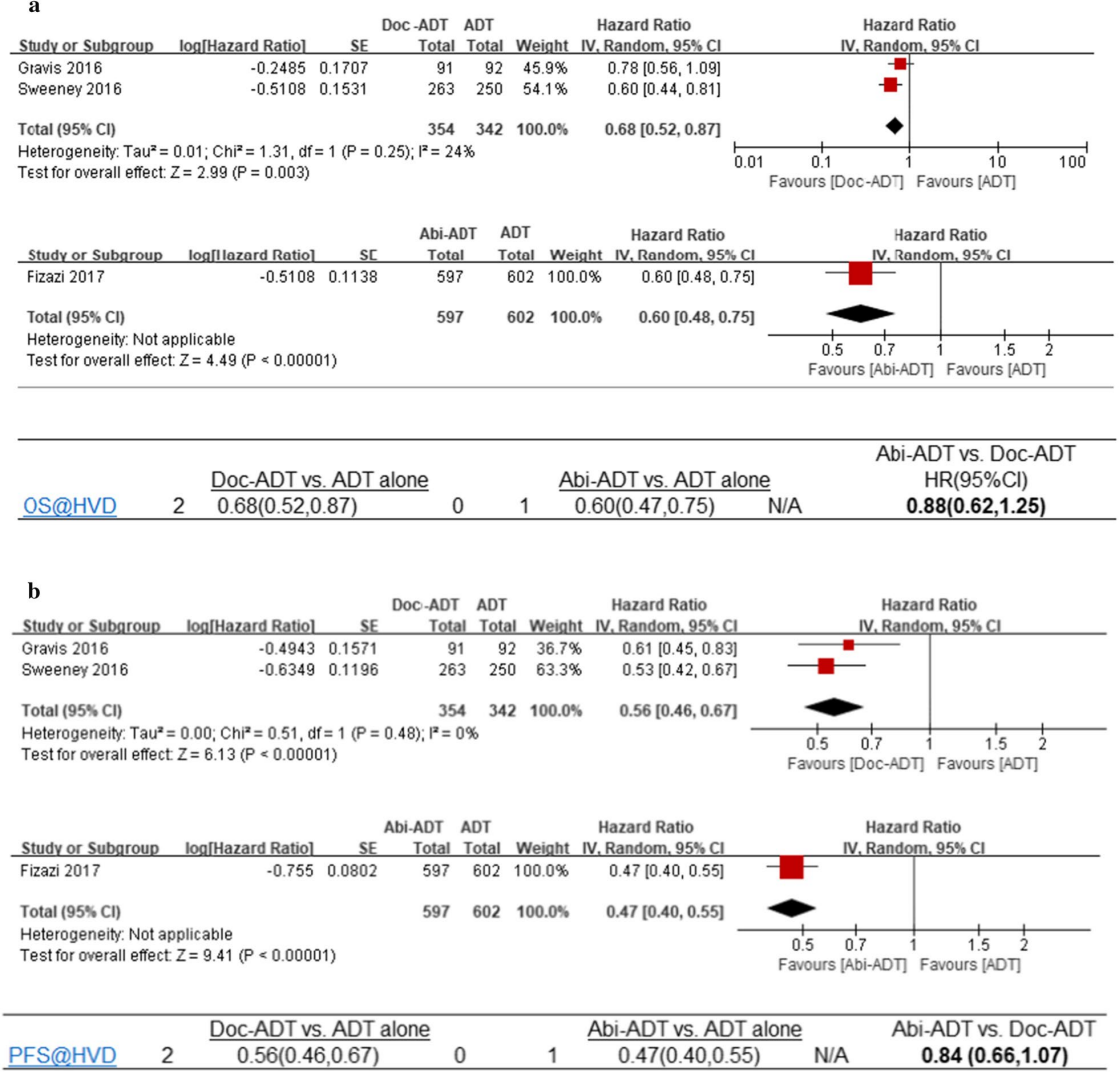
Forest plot for the meta-analysis of combination therapy versus ADT alone with respect to: **a** overall survival (OS) and **b** progression-free survival (PFS). *Abi* abiraterone acetate, *CI* confidence interval, *df* degree of freedom, *Doc* docetaxel, *HR* hazard ratio, *IV* instrumental variables, *SE* standard error

The Wallis study conducted subgroup analyses of OS results of patients with high-volume mHSPC. In our study, we used the same methods for subgroup analyses and obtained equivalent but more detailed results. The pooled HRs assessing OS among patients with high-volume mHSPC were 0.68 (95% confidence interval [CI] 0.52–0.87, *I*^*2*^= 24%, heterogeneity Chi square p = 0.25, 2 trials, 696 patients) for Doc-ADT versus ADT alone and 0.60 (95% CI 0.48–0.75, 1 trial, 1199 patients) for Abi-ADT versus ADT alone (Fig. 2a). The indirect treatment comparison of Abi-ADT versus Doc-ADT indicated no significant difference in OS (HR: 0.88, 95% CI 0.62–1.25; Fig. 2a).

#### PFS

The pooled HRs assessing PFS among patients with high-volume mHSPC were 0.56 (95% CI 0.46–0.67, *I*^*2*^= 0%, heterogeneity Chi square p = 0.48, 2 trials, 696 patients) for Doc-ADT versus ADT alone and 0.47 (95% CI 0.40–0.55, 1 trial, 1199 patients) for Abi-ADT versus ADT alone (Fig. 2b). The indirect comparison of Abi-ADT versus Doc-ADT indicated no significant difference in PFS (HR: 0.84, 95% CI 0.66–1.07; Fig. 2b).

#### Cost analysis

The above results combined with those of the Wallis study demonstrated no significant differences in OS and PFS between patients with high-volume mHSPC treated with Doc-ADT and those treated with Abi-ADT. Based on the equivalent efficacy between Doc-ADT and Abi-ADT, we conducted a cost analysis to compare the local drug and medical costs between these two therapies in China.

### Model overview

A decision-analytic model was built to simulate the disease process of mHSPC and estimate the comparative costs of Doc-ADT and Abi-ADT treatment regimens in a Chinese setting.

Patients with mHSPC were included in the model. Based on disease progression, we defined three health states in the model: progression-free survival (PFS), progressed disease (PD) and death. The model began with 1000 patients. At any given time, a patient would be in one of the three states. A patient could remain in PFS (or PD) or advance to PD (or death) during each cycle, and the patient sample was followed until death. The length of each cycle was 1 month. Direct medical costs were considered, including the costs of the drugs (docetaxel, abiraterone, and prednisolone), hospitalisation costs for chemotherapy, ADT cost per month, laboratory test costs, and costs associated with adverse events (AEs). All costs were discounted 3% annually. Model development and the data analysis were conducted using Microsoft^®^ Excel 2013 (Microsoft, Redmond, WA, USA).

### Model input

**Fig. 3:**
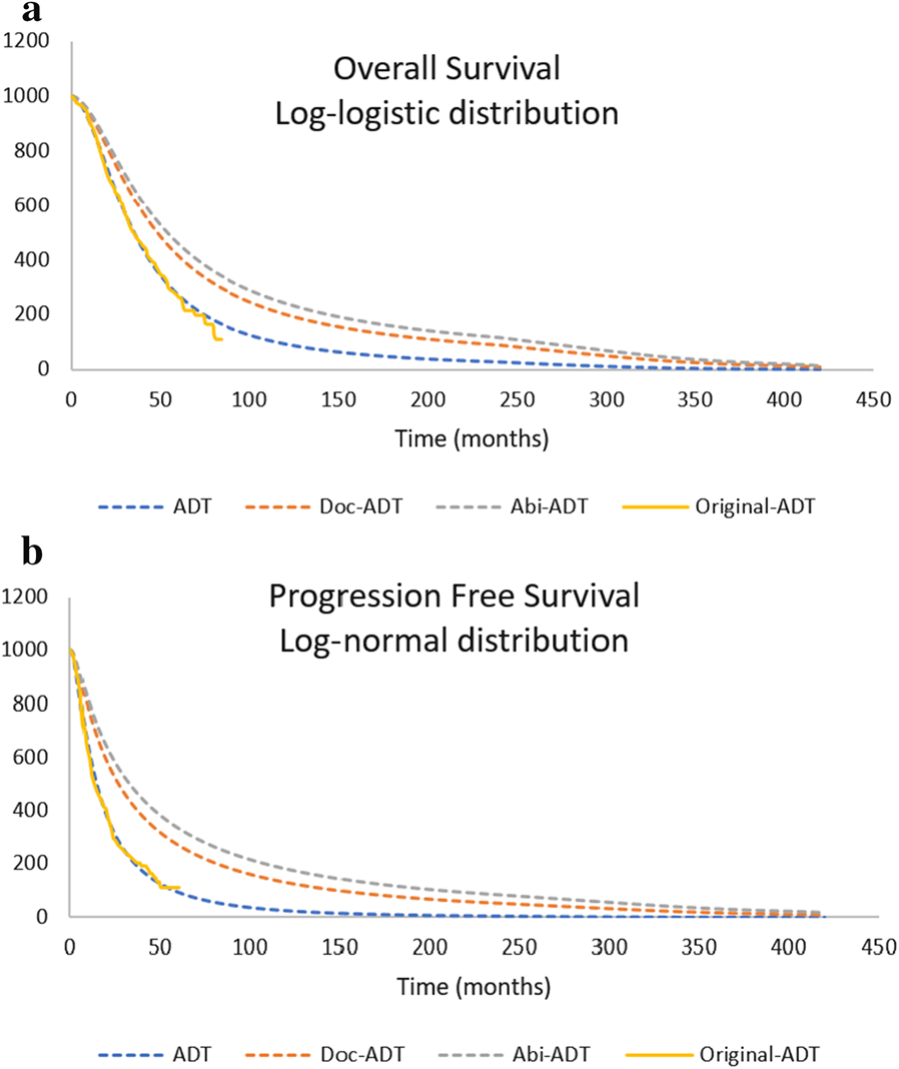
Parametric extrapolation of OS and PFS-Model Base-case with Log-logistic and Log-normal distribution

The numbers of surviving patients, patients with clinical progression and deceased patients each month were obtained by fitting OS and PFS survival curves of the CHAARTED trials ADT arm. Parametric functions were assessed based on model goodness of fit using Akaike Information Criterion (AIC) and Bayesian Information Criterion (BIC) as well as a visual assessment of each parametric function. Log-logistic and Log-normal distribution were selected for the OS and PFS curve respectively based lowest AIC and BIC value. Then, the HRs from NMA were applied to reconstruct the curve for Abi-ADT and Doc-ADT arms (Fig. 3).

For patients with clinical progression, the second-line treatment varied widely across the two therapies. Based on the Sweeney 2015 study [5], 54 of 180 (34%) patients with progression receiving Doc-ADT therapy continued docetaxel treatment, while the remaining patients (66%) were switched to abiraterone. According to Chinese experts’ opinions, the clinical protocols for patients with progression in China are similar. Among the patients receiving Abi-ADT therapy, all patients with progression were switched to docetaxel treatment for 10 cycles.

The unit costs of docetaxel and prednisolone/prednisone were extracted from the IQVIA China Hospital Pharmaceutical Audit (CHPA) database, which was updated in the first three quarters of 2017. Because abiraterone acetate has been included in the national negotiation drug list in China, we used the negotiated price as its drug cost. Weighted average dosages from the included trials were calculated and entered into the cost analysis. Body surface area (BSA, cm^2^) was calculated as 0.0061 × height (cm) + 0.0128 × weight (kg) − 0.1529. An average height of 161.5 cm and an average weight of 61.8 kg, reflecting a typical Chinese adult [18], were suggested by local clinical experts (Table 2).

**Table 2:** Model inputs

Parameters	Base case value	Range tested (%)
Unit drug costs (RMB)		
Unit cost of docetaxel (cost per mg)	73.62	± 20
Unit cost of abiraterone (cost per mg)	0.58	± 20
Unit cost of prednisolone/prednisone (cost per mg)	1.33	± 20
Dosage		
Daily dosage of docetaxel (mg/m^2^)	75	± 20
Daily dosage of abiraterone (mg)	1000	± 20
Daily dosage of prednisolone (mg)	5	± 20
Average height (m)	1.615	± 20
Average weight (kg)	61.8	± 20
Other medical costs (RMB)		
Hospitalisation costs for chemotherapy	318	± 20
Preventive drugs for chemotherapy	620	± 20
ADT cost per month	2050	± 20
Laboratory test costs	500	± 20
Inpatient OOP	15	± 20
Outpatient OOP %	25%	± 20
Costs of adverse events (RMB)		
Hypertension	98.70	± 50
Hypokalaemia	65.00	± 50
ALT increased	225.68	± 50
Hyperglycaemia	115.00	± 50
AST increased	225.68	± 50
Bone pain	583.00	± 50
Cardiac disorder	1000.00	± 50
Anaemia	271.70	± 50
Back pain	73.60	± 50
Spinal-cord compression	1235.00	± 50
Fatigue	540.44	± 50
Allergic reaction	75.00	± 50
Diarrhoea	31.44	± 50
Stomatitis	74.00	± 50
Neuropathy	375.84	± 50
Thromboembolism	1794.20	± 50
Sudden death	3200.00	± 50
Thrombocytopenia	3518.04	± 50
Neutropaenia	720.80	± 50
Febrile neutropaenia	1787.20	± 50
Infection with neutropaenia	14,764.05	± 50

In addition to the costs of docetaxel, prednisolone/prednisone and abiraterone acetate associated with Doc-ADT and Abi-ADT therapies, interviews with clinicians from five major cities in China (Beijing, Shanghai, Wuhan, Chengdu and Guangzhou) were conducted to collect information regarding other medical resource use and cost data related to the two therapies. Other costs included the hospitalisation costs for chemotherapy, ADT cost per month, laboratory test costs, and the costs associated with AEs. Patients receiving docetaxel required hospitalisation, while patients treated with abiraterone acetate did not. The hospitalisation costs for patients receiving docetaxel included hospital bed fees and the cost of preventive drugs for chemotherapy. All patients required regular examinations, including routine blood tests, blood biochemistry and liver function tests. The incidence of AEs was obtained from the included clinical trials and is listed in the Table 3.

**Table 3:** Costs and probability of adverse events

	Abi-ADT		Doc-ADT		Cost used in the model
Hypertension	121	20.3%			98.7
Hypokalaemia	62	10.4%			65.0
Increased ALT	33	5.5%			225.7
Hyperglycaemia	27	4.5%			115.0
Increased AST	26	4.4%			225.7
Bone pain	20	3.4%			73.6
Cardiac disorder	22	3.7%			1000.0
Anaemia	15	2.5%	5	1.3%	271.7
Back pain	14	2.3%			73.6
Spinal-cord compression	12	2.0%			1235.0
Fatigue	10	1.7%	16	4.1%	540.4
Allergic reaction			8	2.1%	75.0
Diarrhoea			4	1.0%	31.4
Stomatitis			2	0.5%	74.0
Neuropathy			4	1.0%	375.8
Thromboembolism			3	0.8%	1794.2
Sudden death			1	0.3%	3200.0
Thrombocytopenia			1	0.3%	3518.0
Neutropaenia			47	12.1%	720.8
Febrile neutropaenia			24	6.2%	1787.2
Infection with neutropaenia			9	2.3%	14,764.1

### Perspectives

We compared the cost between Doc-ADT and Abi-ADT therapies from both healthcare and patient perspectives.

From the healthcare perspective, we calculated the total medical costs of Doc-ADT and Abi-ADT therapies. From the patient perspective, only patient co-payments were considered. Under the current insurance policy (coverage for urban employees and urban and rural residents), using Shanghai as a reference, an average patient co-pay rate of 25% was applied for outpatients, including all the costs associated with Abi-ADT treatment, and 15% for inpatients, including all the costs associated with Doc-ADT treatment.

### Sensitivity analysis

Uncertainty is usually associated with input parameter values of an economic model, which may derive from clinical trials, observational studies or in some cases, expert opinions. Therefore, sensitivity analyses were performed to examine the stability and robustness of the results.

One-way sensitivity analysis (OWSA) was performed for all parameters. The results were recalculated by adjusting one input (parameter) at a time to determine how the model results were affected. The input was specified as multiple point estimates and was adjusted manually. Most parameters included in the one-way sensitivity analyses were adjusted by ± 20%, and the cost data for AEs derived from clinician interviews were adjusted by ± 100%. Because the drug cost of abiraterone used was the negotiated price, its future price cannot be increased. Therefore, only the lower limited unit cost of abiraterone acetate was considered in this analysis. The OWSA results are displayed in a tornado diagram.

To quantify the level of uncertainty in the output of the analysis, we also conducted a probabilistic sensitivity analysis (PSA). In the PSA, 1000 Monte Carlo simulations were performed to test the effect of uncertainty on the base case results for costs. Model input was specified as a distribution and was obtained from the literature. The results are displayed in a histogram for the frequency distribution of cost differences.

### Cost analysis results

#### Healthcare perspective

The expected total costs were RMB 399,844 per patient for Doc-ADT therapy and RMB 1,286,900 per patient for Abi-ADT therapy, with a potential saving of up to RMB 887,057 per patient. Especially, the expected drug costs before clinical progression were RMB 130,786 per patient for Doc-ADT therapy, which is much lower than that for Abi-ADT therapy with RMB 1,230,951 per patient. Although the second-line treatment costs after clinical progression for Doc-ADT therapy is higher than those for Abi-ADT therapy, the total cost for Doc-ADT arm is still much lower (Table 4).

**Table 4:** Base case results from the healthcare perspective and patient perspective (unit: RMB)

	Doc-ADT	Abi-ADT	Difference
Healthcare perspective			
Drug + ADT costs	¥130,786	¥1,230,951	(¥1,100,165)
Medical costs	¥29,098	¥31,339	(¥2,241)
AE costs	¥266	¥1,459	(¥1,193)
2nd-line Tx costs	¥239,694	¥23,152	¥216,542
Total	¥399,844	¥1,286,900	(¥887,057)
			Cost saving
Patient perspective			
Drug + ADT costs	¥27,611	¥307,738	(¥280,127)
Medical costs	¥6,459	¥7,835	(¥1,376)
AE costs	¥40	¥365	(¥325)
2nd-line Tx costs	¥59,091	¥3,473	¥55,618
Total	¥93,200	¥319,410	(¥226,210)
			Cost saving

**Fig. 4:**
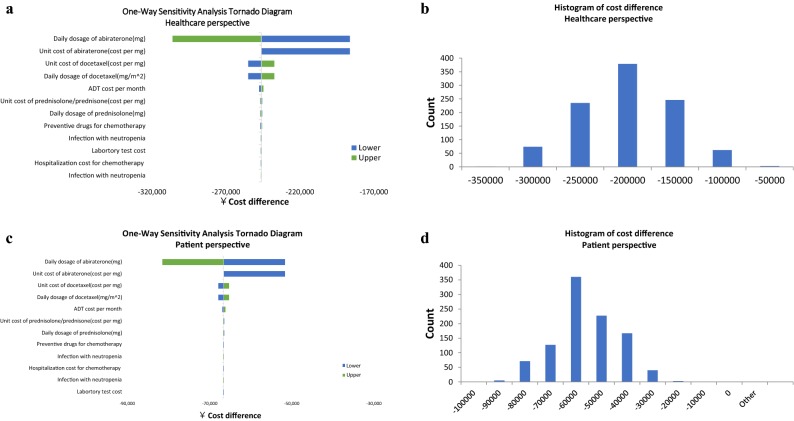
**a** Tornado diagram for the one-way sensitivity analysis of the cost analysis (healthcare perspective); **b** Histogram of the lifetime cost difference for the probabilistic sensitivity analysis (healthcare perspective); **c** Tornado diagram for the one-way sensitivity analysis of the cost analysis (patient perspective); **d** Histogram of the lifetime cost difference for the probabilistic sensitivity analysis (patient perspective)

The results of the OWSA and PSA are displayed in the tornado diagram and histogram presented in Fig. 4a, b. The variables with the greatest impact on the cost difference were dosage and the unit cost of abiraterone acetate. In the PSA, Doc-ADT therapy was a cost-saving alternative in 100% of the simulations.

#### Patient perspective

The expected total out-of-pocket costs were RMB 93,200 per patient for Doc-ADT therapy and RMB 319,410 for Abi-ADT therapy, with a potential saving of up to RMB 226,210 per patient (Table 4). The results of OWSA and PSA are displayed in Fig. 4c, d).

## Discussion

The network meta-analysis of Wallis et al. [19] demonstrated no significant difference in OS for patients with mHSPC treated with Doc-ADT versus Abi-ADT therapy. However, the pooled result of PFS was not reported. Our study provided an update of the Wallis study for patients with high-volume mHSPC. We used the same search strategy and extended the search period to November 2017. One additional paper reporting the PFS results from the CHAARTED trial was included. Our study found no significant difference in PFS between the two therapies.

Besides clinical effects, treatment cost is another crucial factor in choosing treatment strategies in China. Therefore, we conducted an economic evaluation to compare the costs of Doc-ADT and Abi-ADT therapies in China during the entire course of disease progression until patient death, based on their equivalent clinical effects. Our study demonstrated that Doc-ADT therapy was a cost-saving alternative to Abi-ADT therapy in Chinese patients, with potential savings of up to RMB 887,057 per patient from the healthcare perspective and RMB 226,210 per patient from the patient perspective. Sensitivity analyses confirmed the robustness and steadiness of these results.

Similar cost analyses of prostate cancer treatments had been assessed from the perspective of multiple countries in past few years. The STAMPEDE trial [20] in the UK compared the costs and quality of life associated with Doc-ADT and ADT therapies among men and reported that adding docetaxel to ADT was estimated to extend QALYs by 0.51 years in M1 patients (the cancer has spread to distant sites) and 0.39 years in M0 patients (no growth to distant sites). This finding demonstrated that adding the chemotherapeutic agent docetaxel to standard hormone therapy for mHSPC improves patients’ overall quality of life (QoL), reduces the need for subsequent therapy, and is cost-saving. The LATITUDE study [21] compared patient-reported outcomes following Abi-ADT therapy with ADT alone in patients with newly diagnosed metastatic castration-naive prostate cancer and reported that Abi-ADT improves patients’ QoL. There are also several studies assess the cost for abiraterone or docetaxel in metastatic castration-resistant prostate cancer (mCRPC) [20, 21], however, no study have been performed regarding with the head-to-head comparisons between Doc-ADT and Abi-ADT among patients with mHSPC.

One of the strengths of our analysis is that all cost data were collected from published literature and confirmed by local clinical experts or were directly provided by experts, reflecting current costs as well as current clinical practices in China. Furthermore, we broke down lifetime costs even beyond the clinical trial period for Doc-ADT and Abi-ADT therapies, which are presented in Table 3. For Doc-ADT therapy, we adopted the second-line treatment cost after clinical progression used in the CHAARTED trial (34% patients continued Doc-ADT, and 66% were switched to Abi-ADT). For Abi-ADT therapy, since no suggestions were identified in the included trials, suggestions from local clinical experts were applied; accordingly, all patients were switched to Doc-ADT for 10 cycles. Therefore, although Doc-ADT treatment is less expensive than Abi-ADT treatment, the second-line treatment cost after clinical progression associated with the former is greater than that associated with the latter. Another strength of our analysis is that we extrapolated the PSF and OS of CHAARTED trials ADT arm survival curve to a lifetime horizon, and then applied HRs from the indirect comparison to reconstruct the curve for Abi-ADT and Doc-ADT arms. In this case, the syntheses of evidence of indirect comparison were used.

Some limitation should be noted. First, although the method of indirect comparison was validated for comparing outcomes for RCTs, the approach is still a surrogate. Head-to-head trial comparing Doc-ADT and Abi-ADT was recommended in the future. Second, the incidence of AE for Doc-ADT and Abi-ADT were collected from the corresponding arms in the CHAARTED and LATITUDE trials, respectively. We have conducted clinician’s interview, and the clinicians confirmed that there were minor differences in follow-up time and baseline characteristics between the two studies, which had little impact on the results of the cost analysis. Based on the results in the OWSA, the incidence of AEs had little effect on the results after we tested a broad range of adjustments. Third, because the patient co-pay rates for outpatients and inpatients vary across different provinces in China, we used the average patient co-pay rates in Shanghai as a reference for the patient perspective as suggested by local clinicians. Nevertheless, the sensitivity analysis verified the stability and robustness of the results.

## Conclusion

Our indirect comparison shows no significant difference in either PFS or OS between these two therapies in the treatment of patients with high-volume mHSPC. The cost analysis demonstrates that Doc-ADT therapy is a cost-saving alternative to Abi-ADT in China.

## Summary points

- The objective of this paper is to conduct an indirect treatment comparison of patients with high-volume mHSPC and a cost analysis between Abi-ADT and Doc-ADT therapies in China.
- The Bucher technique for indirect treatment comparison was used to compare PFS in patients treated with Abi-ADT and those treated with Doc-ADT.
- Overall, 1895 patients from three trials reporting PFS data were included; 354 (18.7%) patients received Doc-ADT, 597 (31.5%) patients received Abi-ADT, and 944 (49.8%) patients received ADT alone.
- The indirect treatment comparison demonstrated no significant difference in PFS for Abi-ADT versus Doc-ADT (HR: 0.84, 95% CI 0.66–1.07).
- A cost analysis over a lifetime survival projection was conducted based on the updated systematic review and indirect comparison. The related medical cost was derived from the local setting.
- The cost analysis indicated that Doc-ADT therapy costs less than Abi-ADT, with potential savings of up to RMB 887,057 per patient from the healthcare perspective and RMB 226,210 per patient from the patient perspective.
- No significant differences in either PFS or OS exist between Doc-ADT and Abi-ADT therapy for patients with high-volume mHSPC. The cost analysis revealed that Doc-ADT therapy is a cost-saving alternative to Abi-ADT in China.

## Data Availability

Data sharing is not applicable to this article as no datasets were generated or analysed during the current study.
